# Sequence characterization, molecular phylogeny reconstruction and recombination analysis of the large RNA of *Tomato spotted wilt virus* (*Tospovirus*: *Bunyaviridae*) from the United States

**DOI:** 10.1186/s13104-016-1999-1

**Published:** 2016-04-01

**Authors:** Shunmugiah V. Ramesh, Hanu R. Pappu

**Affiliations:** Department of Plant Pathology, Washington State University, 123 Vogel Plant BiologicalSciences, Pullman, WA 99164 USA; ICAR-Directorate of Soybean Research, Khandwa Road, Indore, 452 001 Madhya Pradesh India

**Keywords:** Genetic diversity, Recombination, RNA dependent RNA polymerase, *Tomato spotted wilt virus*, *Tospovirus*

## Abstract

**Background:**

*Tomato spotted wilt virus* (TSWV; *Tospovirus*: *Bunyaviridae*) has been an economically important virus in the USA for over 30 years. However the complete sequence of only one TSWV isolate PA01 characterized from pepper in Pennsylvania is available.

**Results:**

The large (L) RNA of a TSWV WA-USA isolate was cloned and sequenced. It consisted of 8914 nucleotides (nt) encoding a single open reading frame of 8640 nts in the viral-complementary sense. The ORF potentially codes for RNA-dependent RNA polymerase (RdRp) of 330.9 kDa. Two untranslated regions of 241 and 33 nucleotides were present at the 5′ and 3′ termini, respectively that shared conserved tospoviral sequences. Phylogenetic analysis using nucleotide sequences of the complete L RNA showed that TSWV WA-USA isolate clustered with the American and Asian TSWV isolates which formed a distinct clade from Euro-Asiatic Tospoviruses. Phylogeny of the amino acid sequence of all tospoviral RdRps used in this study showed that all the known TSWV isolates including the USA isolate described in this study formed a distinct and a close cluster with that of *Impateins necrotic spot virus*. Multiple sequence alignment revealed conserved motifs in the RdRp of TSWV. Recombination analysis identified two recombinants including the TSWV WA-USA isolate. Among them, three recombination events were detected in the conserved motifs of the RdRp.

**Conclusions:**

Sequence analysis and phylogenetic analysis of the L RNA showed distinct clustering with selected TSWV isolates reported from elsewhere. Conserved motifs in the core polymerase region of the RdRp and recombination events were identified.

**Electronic supplementary material:**

The online version of this article (doi:10.1186/s13104-016-1999-1) contains supplementary material, which is available to authorized users.

## Background

Tospoviruses (family *Bunyaviridae*, genus *Tospovirus*) are important pathogens that cause considerable economic losses to field and horticultural crops worldwide [1, 2]. Tospoviruses are transmitted by thrips (*Thysanoptera*, *Thripidae*) in a persistent and circulative manner [3]. *Tomato spotted wilt virus* (TSWV) is one of more than 29 known Tospoviruses, and is considered as one of the top ten important plant viruses worldwide [4]. The TSWV virions are quasi-spherical and are 80–100 nm in diameter. The TSWV genome comprises of three single-stranded RNAs that are individually encapsidated by a nucleoprotein and collectively packaged inside a glycoprotein envelope. The tripartite genome of TSWV is characterized by Large (L), Medium (M) and Small (S) RNAs. The L RNA segment encodes RNA dependent RNA polymerase (RdRp) in the viral complementary sense [5]. The distinctive feature of Tospoviruses is the presence of ambisense strategy in the S and M genomic segments. The S and M genomic RNAs code for the non-structural proteins (NSs and NSm) respectively in the viral sense, whereas the N and G_N_/G_C_ are coded for in the viral complementary sense [6–8].

Despite the importance of TSWV as one of the most widely prevalent and persistent viral pathogens in the US, sequence information of the complete L RNA segment from the US is available for one TSWV PA01 isolate characterized from pepper in Pennsylvania [9]. The virion packages a few molecules of RdRp which ensures transcription of virion RNA to positive sense (translatable RNA) during early stages of virus infection. Thus, the tospoviral RdRp is also referred to as L-protein and performs many conserved functions with regard to virus genome replication inside the host cell. Moreover, the RdRp-encoding regions of plant pathogenic viruses have been shown to display considerable genetic variability and recombination events [10–12]. A recent report of the broad spectrum transgenic resistance against several Tospoviruses made use of the conserved tospoviral RdRp gene sequences [13]. Here we report the complete nucleotide sequence features of L RNA of a TSWV isolate from the USA, and searched for the conserved amino acid sequences in the core polymerase region of RdRp and potential recombination events. Results of the phylogenetic analysis of L RNA of TSWV, its RdRp, along with identification of recombination events and their implications are also discussed.

## Methods

### Virus source, RNA extraction, RT-PCR and sequencing

**Fig. 1:**
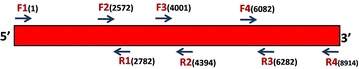
Scheme depicting TSWV L-RNA, and primers co-ordinates in L-RNA [Complete L RNA of TSWV WA-USA isolate was amplified as overlapping fragments by designing primers (*F* forward primers; *R* reverse primers) based on the genome sequences available in the GenBank database]

The TSWV WA-USA was maintained on tomato plants under controlled greenhouse conditions. Total RNA was extracted from infected leaves using TRIzol reagent (Invitrogen, USA) following the manufacturer’s protocol and was used for cDNA synthesis using primer pairs listed in Table 1. Oligonucleotides were designed based on the sequences available in GenBank and were used to amplify the complete L RNA segment of TSWV as overlapping fragments (Fig. 1). Following RT-PCR, the resulting overlapping amplicons were cloned into pGEMT-Easy (Promega, Madison, USA). Recombinant clones were selected and the plasmid DNA was prepared and sequenced (ELIM Biopharma, Hayward, USA). At least three clones were sequenced for each of the virus genome fragments that were cloned as overlapping fragments.

**Table 1:** Primers used for the amplification of the large (L) RNA of Tomato spotted wilt virus WA-USA (TSWV WA-USA) isolate

Oligonucleotides	Sequence (5′-3′)	Position in L RNA	Expected ampliconsize (bp)
TSWVF1	AGAGCAATCAGGTAACAACG	1	2782
TSWVR1	AATCGGACAGTGGCAAAAAC	2782	
TSWVF2	GCGCAATTTTCAAAGAAAGC	2572	1822
TSWVR2	AACCAGTTCATGCTAACAGG	4394	
TSWVF3	CAGAAGCCATATCTATAAGTGG	4001	2281
TSWVR3	GGTGGATAGGAGAGCCAATG	6282	
TSWVF4	GGCAAAGACAGCTTCGAGAC	6082	2832
TSWVR4	AGAGCAATCAGGTACAACTAAAAC	8914

### Sequence annotation and analysis

The complete L RNA genomic component of TSWV WA-USA isolate was assembled and reconstructed from the overlapping clones using Bio-Edit sequence alignment editor software [14]. Pair-wise and multiple alignments were done using CLUSTAL-W in MEGA 6 [15]. Phylogenetic tree was drawn using the maximum-likelihood statistical method based on the Tamura-Nei model [16]. The test of phylogeny was performed by bootstrap method with 1000 bootstrap replications.

### Detection of recombination

Recombination Detection Program-4 (RDP 4 Beta 4.27) [17] was used to identify potential recombination events among the L RNA sequences of the Tospoviruses used in this study. Recombination detection analysis was performed with default settings for all the methodologies available in RDP 4 [17], however, in the recombination detection option, the highest acceptable *p* value was at 0.01.

## Results and discussion

### Complete L RNA segment of TSWV WA-US isolate

The complete L RNA of TSWV WA-USA isolate was 8914nts in length [GenBank Accession no. KP827649] and encodes an ORF of 8640 nts (from nt position 242 through 8793) in viral complementary sense. The deduced amino acid sequence of the ORF is 2879 in length and is predicted to code for a 330.9 kDa protein. The comparative sequence analysis of the ORF and amino acid sequence showed that this protein is an RNA dependent RNA polymerase (RdRp). The TSWV WA-USA isolate shared highest nt identity (98 %) with the known TSWV isolates reported from Japan (AB198742), Korea (HM581940; HM581937; AB190813) and Spain (KP008132). Further TSWV WA-USA isolate shared 97 % nt identity with another TSWV US isolate PA01 (KT160280). A 241 and 33 nt untranslated region (UTR) was identified at the 5 and 3′ ends of the L segment respectively. These UTRs shared the first 16 nt (agagcaatcaggtaac) that are conserved terminal sequences for *Tospovirus* genome segments [2].

### Molecular phylogeny

**Fig. 2:**
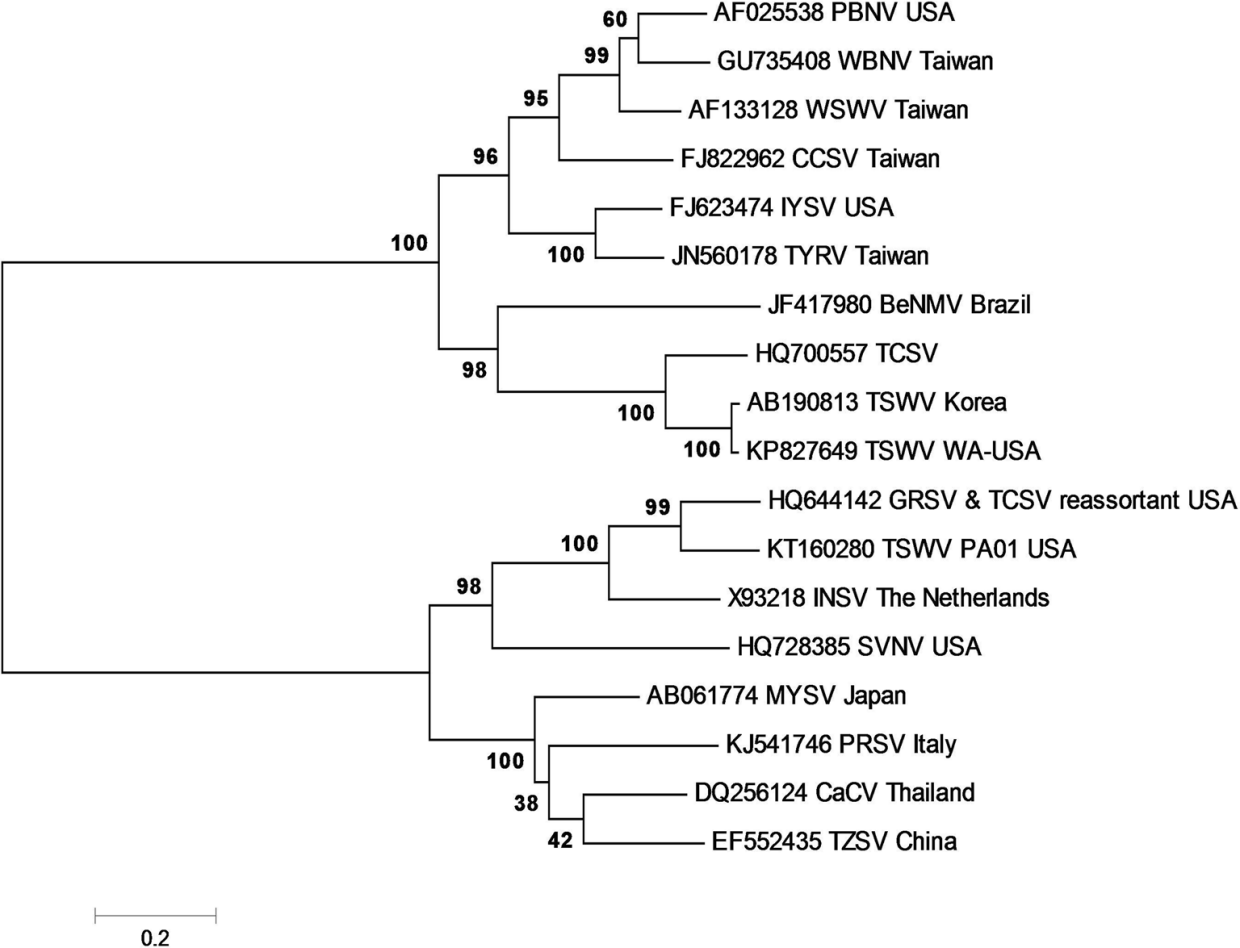
Phylogenetic analysis of the complete nucleotide sequences of the large (L) RNA of known Tospoviruses by maximum likelihood method. Bootstrap values on the branches represent the percentages out of 1000 bootstrap replicates. The data matrix containing Tospovirus L RNA sequence alignment and phylogenetic analyses were deposited into TreeBase under accession url (http://purl.org/phylo/treebase/phylows/study/TB2:S18894). The list of *Tospovirus* sequences and their abbreviations are provided in Additional file 1: Table S1

The complete L RNA sequences of known Tospoviruses were analyzed to study its molecular phylogeny. The evolutionary history was inferred by using the maximum likelihood method based on the Tamura-Nei model (Fig. 2). The phylogenetic tree showed that all known L RNA sequences of Tospoviruses formed two distinct clades that consisted of sub-clades (Fig. 2). The L RNA sequence of the TSWV WA-USA isolate was within one major clade consisting of other American and Asiatic isolates [Peanut bud necrosis virus (PBNV) (AF025538), Watermelon bud necrosis virus (WBNV)(GU735408), Watermelon spotted wilt virus (WSWV) (AF133128), Calla lily chlorotic spot virus (CCSV) (FJ822962), Iris yellow spot virus (IYSV) (FJ623474), Tomato yellow ring spot virus (TYRV) (JN560178), Bean necrotic mosaic virus (BeNMV) (JF417980),and Tomato chlorotic spot virus (TCSV) (HQ700667)]. Within this clade, TSWV isolates formed a distinct sub-clade along with TCSV (HQ700667) and BeNMV (JF417980) suggesting its distinct lineage. The other major clade included Tospoviruses primarily of Euro-Asiatic origin (KJ541746 PolRSV Italy, X93218 INSV-The Netherlands, DQ256124 CaCV-Thailand, AB061774 MYSV-Japan, and EF552435, TZSV-China) along with an American *Tospovirus* that was a result of genetic re-assortment between GRSV and TCSV (HQ644142), SVNV isolate from the USA (HQ728385) and another TSWV isolate (PA01) characterized from pepper reported from USA [9].

**Fig. 3:**
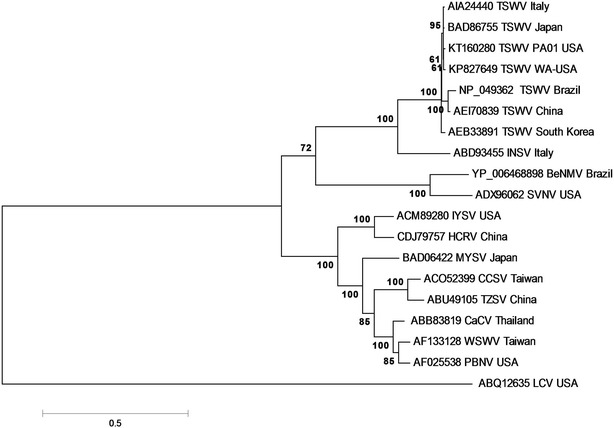
Phylogenetic analysis based on the deduced amino acid sequences of the RNA dependent RNA polymerase (RdRp) of known Tospoviruses by Maximum Likelihood method. Bootstrap values on the branches represent the percentages out of 1000 bootstrap replicates. The tree is rooted on La crosse virus (ABQ12635) as an outgroup. The data matrix containing *Tospovirus* RdRp sequence alignment and phylogenetic analyses were deposited into TreeBase under accession url (http://purl.org/phylo/treebase/phylows/study/TB2:S18894). The list of *Tospovirus* sequences and their abbreviations are provided as Additional file 1: Table S2

Molecular phylogenetic relationships of RdRp amino acids sequences were inferred employing maximum-likelihood statistical method. Phylogeny reconstruction revealed that the RdRp encoded by TSWV WA-USA isolate was part of a group consisting of other TSWV isolates (Fig. 3). The phylogenetic study of RdRp sequences showed the presence of three distinct clades consistent with the distinct evolutionary lineages proposed for Tospoviruses, SVNV and BeNMV [18]. Phylogenetic analysis of the complete L RNA nucleotide sequences showed the presence of only two distinct lineages in which BeNMV clustered with TSWV isolates and SVNV clustered with Tospoviruses belonging to the Euro-Asiatic group (Fig. 2).

### Conserved RdRp motifs

**Fig. 4:**
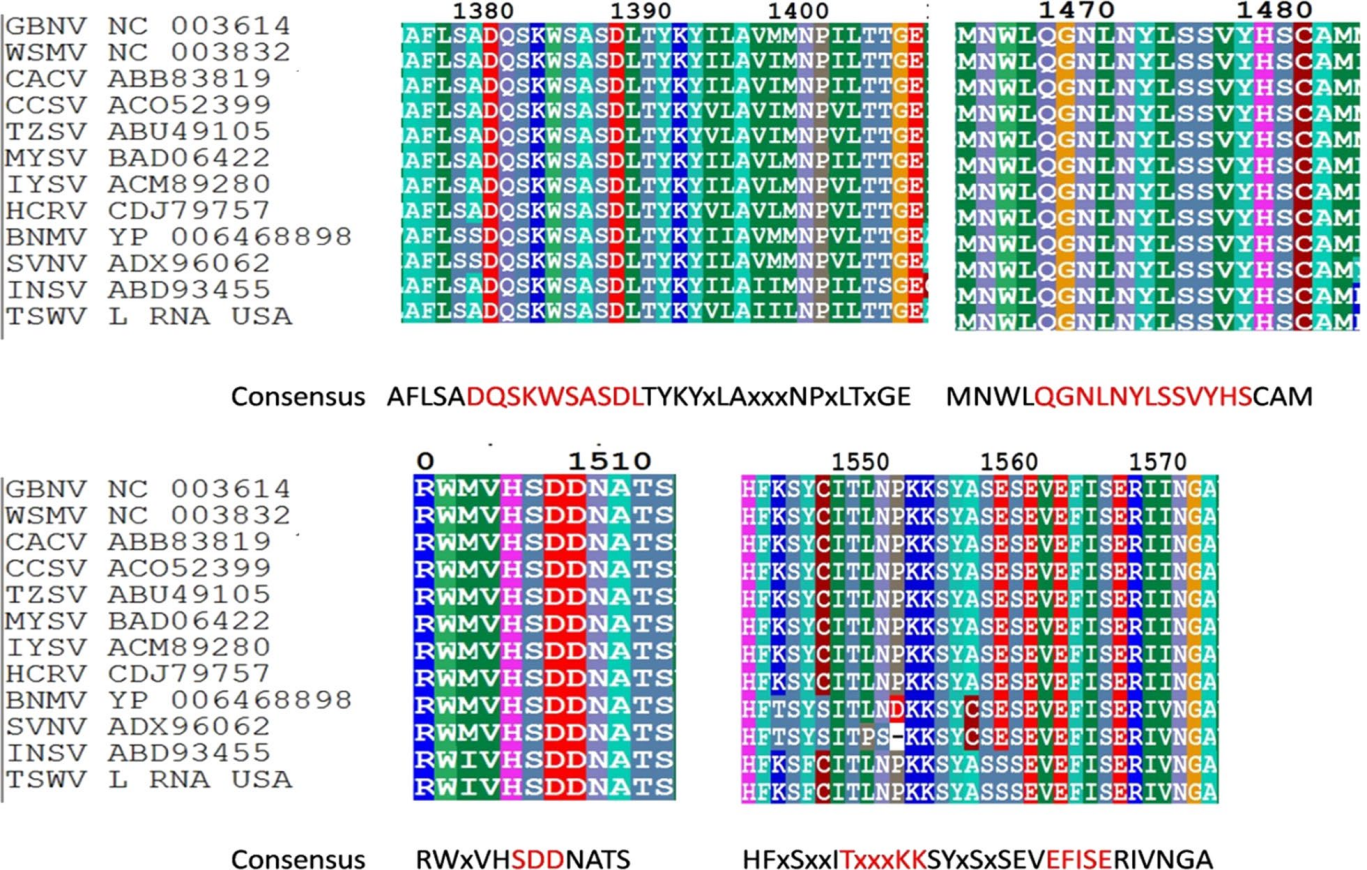
Conserved motifs in RNA-dependent RNA polymerase (RdRp) of Tospoviruses. The consensus sequences of the motifs are highlighted in *red*

Sequence analysis of the core polymerase region of RdRp of the TSWV WA-USA isolate showed the presence of all the five conserved motifs (Fig. 4) characteristic of *Tospovirus* RdRps. These include motif A (DxxKWS), motif B (QGxxxYxSS), motif C (SDD), motif D (TxxxKK), and motif E (EFxSE) [13, 19]. These conserved regions were used in transgenic research to provide broad spectrum resistance against Tospoviruses [13].

### Recombination detection

**Fig. 5:**
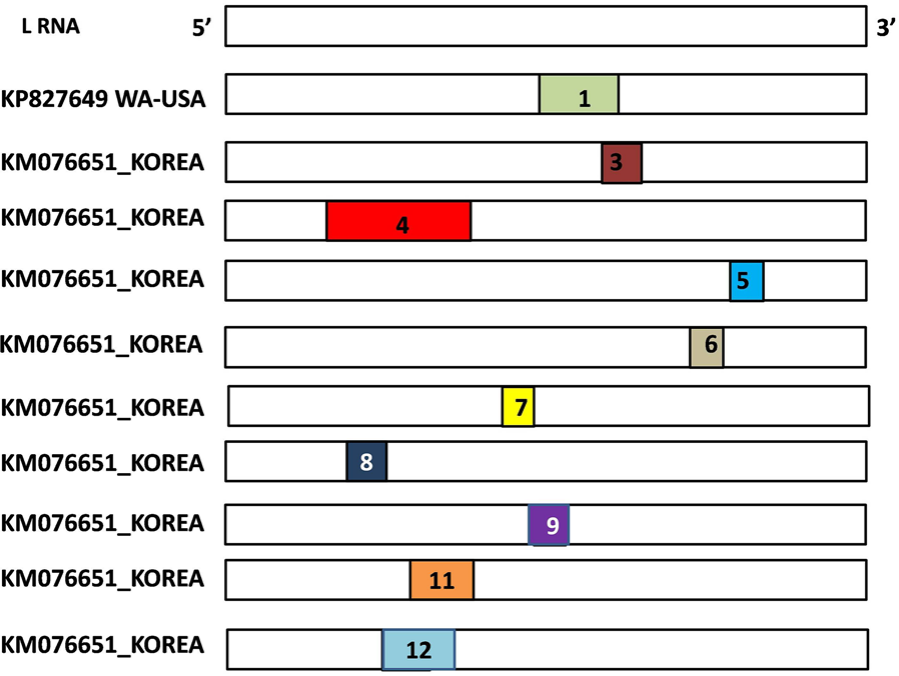
Recombination analysis of the known *Tomato spotted wilt virus* L RNA segment using RDP v 4.27. Among the 23 complete L RNA sequences, two recombinants have been identified (KP827649, KM076651) resulting from ten recombination events. Details of the L RNA isolates, recombination/cross-over region and the parents involved have been described in the Additional file 1: Tables S3, S4

Analysis of the 23 complete L RNA sequences (including that of the TSWV WA-USA and TSWV PA01 isolates) revealed ten recombination events. Details of recombination detection study including the events, major and minor parents involved, beginning and end of break-points are provided in Additional file 1: Table S4. Among the ten recombination events, one event identified isolate TSWV WA-USA as a recombinant arising from the major parent (HM581937), a TSWV-Pepper isolate from South Korea and minor parent (KC261971) another South Korean TSWV isolate (TSWV-17). The event was localized at positions 4534 and 5536 in the alignment (Fig. 5). Further, seven among the nine algorithms used (RDP, Chimaera, BootScan, 3Seq, GENECONV, MaxChi, SiScan and LARD, PhylPro), detected this event except LARD and PhylPro. Moreover, the recently described Korean isolate KM076651, was found to be a recombinant evolved from nine different recombination events (Fig. 5). Among these nine recombination events, three events (Event numbers 1, 7 and 9) that resulted in two putative recombinants (TSWV WA-USA and KM076651) were found to be involved in conserved RdRp motifs (Fig. 4 and Additional file 1: Table S4). Despite recombination events within the conserved RdRp motifs, consensus amino acid sequences in the core polymerase region of RdRp are maintained among various TSWV isolates. This indicates the role of the functional significance of these conserved amino acid sequences in shaping viral genome evolution.

## Conclusions

TSWV continues to be a production constraint to several crops in the US and elsewhere in the world, and with this study, the complete genomic sequence of L RNA of an isolate from the US is now available. Besides providing insights into the molecular phylogeny of the TSWV L RNA genomic segment, we analyzed the role of genetic recombination in the evolution of the L RNA. The results of phylogenetic analysis and recombination studies imply TSWV isolates from Euro-Asiatic region as a potential origin of TSWV USA isolate. Molecular characterization of complete genomes of other TSWV isolates prevalent in USA and their phylogenetic analysis would clearly depict the evolution of USA isolates. Identification of recombination events within the conserved region of RdRp in this study further supports the role of genetic recombination in driving the evolution of TSWV. Conserved motifs found in the core polymerase region of the RdRp would not only be useful for further studies on the genetic diversity and genome evolution, but also on the structure–function studies of the L RNA and the RdRp.

### Availability of data and materials

*Tospovirus* sequence alignment and corresponding phylogeny trees were submitted to TreeBASE (Accession number S18894) which can be accessed from the URL.

[http://purl.org/phylo/treebase/phylows/study/TB2:S18894]. The data sets supporting the results of this article are included within the article and its additional files. The nucleotide sequence features described in this study is accessioned in GenBank, NCBI [Accession no. KP827649].

## Additional file

10.1186/s13104-016-1999-1 Tospovirus L RNA and RdRp sequences used in the phylogeny and recombination detection analysis and RDP 4 Beta 4.27 results.

## Abbreviations

L RNA: Large RNA
nt: nucleotides
ORF: open reading frame
RDP: Recombination Detection Program
RdRp: RNA dependent RNA Polymerase
RT-PCR: reverse transcription-polymerase chain reaction
TSWV: *Tomato spotted wilt virus*
UTR: untranslated region
