# Supplementary material for: Sequence characterization, molecular phylogeny reconstruction and recombination analysis of the large RNA of Tomato spotted wilt virus (Tospovirus: Bunyaviridae) from the United States
Source: BMC Res Notes. 2016 Apr 1;9:200. doi: 10.1186/s13104-016-1999-1 (PMC4818514; doi:10.1186/s13104-016-1999-1)
Supplement: Supplementary file 1 — 10.1186/s13104-016-1999-1 Tospovirus L RNA and RdRp sequences used in the phylogeny and recombination detection analysis and RDP 4 Beta 4.27 results. [file 13104_2016_1999_MOESM1_ESM.docx]

**Supplementary Table S1**: List of the virus isolates used for the phylogenetic analysis, with acronym, complete virus name and accession number of the L RNA genomic segment

| **S.No** | **Accession no.** | **Virus name and acronym** | **origin** |
| --- | --- | --- | --- |
| 1 | KJ541746 | *Polygonum ring spot virus* (PolRSV) | Italy |
| 2 | AF025538 | *Peanut bud necrosis virus* (PBNV) | USA |
| 3 | X93218 | *Impatiens necrotic spot virus* (INSV) | The Netherlands |
| 4 | HQ700667 | *Tomato chlorotic spot virus* (TCSV) | Brazil |
| 5 | AB190813 | *Tomato spotted wilt virus* (TSWV) | Korea |
| 6 | AF133128 | *Water melon spotted wilt virus* (WSWV) | Taiwan |
| 7 | JF417980 | *Bean necrotic mosaic virus* (BeNMV) | Brazil |
| 8 | FJ822962 | *Calla lily chlorotic spot virus* (CCSV) | Taiwan |
| 9 | DQ256124 | *Capsicum chlorosis virus* (CaCV) | Thailand |
| 10 | HQ644142 | *Groundnut ring spot virus* (GRSV) & *Tomato chlorotic spot virus* (TCSV) re-assortment | USA |
| 11 | FJ623474 | *Iris yellow spot virus* (IYSV) | USA |
| 12 | AB061774 | *Melon yellow spot virus* (MYSV) | Japan |
| 13 | HQ728385 | *Soybean vein necrosis virus* (SVNV) | USA |
| 14 | JN560178 | *Tomato yellow ring virus* (TYRV) | Iran |
| 15 | EF552435 | *Tomato zonate spot virus* (TZSV ) | China |
| 16 | GU735408 | *Watermelon bud necrosis virus* (WBNV) | Taiwan |
| 17 | KT160280 | *Tomato spotted wilt virus* (TSWV) ( PA01) | USA |
| 18 | KP827649 | *Tomato spotted wilt virus* (TSWV) ( WA-USA) | USA |

**Supplementary Table S2**: List of the virus isolates used for the phylogenetic analysis, with acronym, complete virus name and accession number of the RdRp protein

| **S.No** | **Accession no.** | **Virus name and acronym** | **origin** |
| --- | --- | --- | --- |
| 1 | NP_049362 | *Tomato spotted wilt virus* (TSWV) | Brazil |
| 2 | AEI70839 | *Tomato spotted wilt virus* (TSWV) | China |
| 3 | AEB33891 | *Tomato spotted wilt virus* (TSWV) | South Korea |
| 4 | AIA24440 | *Tomato spotted wilt virus* (TSWV) | Italy |
| 5 | ABB83819 | *Capsicum chlorosis virus* (CaCV) | Thailand |
| 6 | ACO52399 | *Calla lily chlorotic spot virus* (CCSV) | Taiwan |
| 7 | ABU49105 | *Tomato zonate spot virus* (TZSV) | China |
| 8 | BAD06422 | *Melon yellow spot virus* (MYSV) | Japan |
| 9 | ACM89280 | *Iris yellow spot virus* (IYSV) | USA |
| 10 | CDJ79757.1 | *Hippeastrum chlorotic ringspot virus* (HCRV) | China |
| 11 | YP_006468898 | *Bean necrotic mosaic virus*(BeNMV) | Brazil |
| 12 | ADX96062 | *Soybean vein necrosis virus* (SVNV) | USA |
| 13 | ABD93455 | *Impatiens necrotic spot virus* (INSV) | Italy |
| 14 | BAD86755 | *Tomato spotted wilt virus* (TSWV) | Japan |
| 15 | AF133128 | *Watermelon spotted wilt virus* (WSWV) | Taiwan |
| 16 | AF025538 | *Peanut bud necrosis virus* (PBNV) | USA |
| 17 | KT160280 | *Tomato spotted wilt virus* (TSWV) ( PA01) | USA |
| 18 | KP827649 | *Tomato spotted wilt virus* (TSWV) ( WA-USA) | USA |
| 19 | ABQ12635.1 | *La Crosse virus* (LCV) | USA |

**Supplementary Table S3**: List of the TSWV virus isolates used for the recombination detection analysis, and accession number of the L RNA genomic segment, country of origin and year of report

| **S.No** | **Accession no.** | **Country of origin** | **Year** |
| --- | --- | --- | --- |
| 1 | NC_002052 | Brazil | 2008 |
| 2 | AB190813 | Korea | 2004 |
| 3 | HM581940 | S. Korea Pepper2 CY-CN | 2010 |
| 4 | HM581937 | S. Korea Pepper 1 CY-CN | 2010 |
| 5 | KJ575620 | Italy isolate p105 | 2014 |
| 6 | JF960237 | China TSWV-YN | 2011 |
| 7 | KC261947 | S. Korea TSWV-4 | 2012 |
| 8 | KC261950 | S. Korea TSWV-5 | 2012 |
| 9 | KC261953 | S. Korea TSWV-6 | 2012 |
| 10 | KC261956 | S. Korea TSWV-7 | 2012 |
| 11 | KC261959 | S. Korea TSWV-8 | 2012 |
| 12 | KC261962 | S. Korea TSWV-10 | 2012 |
| 13 | KC261965 | S. Korea TSWV-12 | 2012 |
| 14 | KC261968 | S. Korea TSWV-16 | 2012 |
| 15 | KC261971 | S. Korea TSWV-17 | 2012 |
| 16 | KC261974 | S. Korea TSWV-18 | 2012 |
| 17 | AB198742 | Japan | 2005 |
| 18 | HM581934 | S. Korea Tomato NJ-JN | 2010 |
| 19 | KM076651 | Korea LS3 | 2014 |
| 20 | KJ575619 | Italy p202/3WT | 2014 |
| 21 | JN664254 | China CG-1 | 2011 |
| 22 | KT160280 | TSWV (PA01) | 2015 |
| 23 | KP 827649 | TSWV (WA-USA ) | 2014 |

**Supplementary Table: S4** Results of Recombination detection analysis in complete TSWV L RNA genomic segment using RDP 4 Beta 4.27

| Event | Recombinant | Major parent | Minor parent | Start break point | End break point | Algorithms detecting the event and their respective P-values |
| --- | --- | --- | --- | --- | --- | --- |
| 1 | TSWV WA-USA | HM581937 | KC261971 | 4534 | 5536 | RDP (3.261 E-39), GENECONV(6.074 E-42), BootScan (4.155 E-45), MaxChi (8.402 E-16), Chimaera (7.560 E-16), SiScan (1.015 E-22), 3Seq (1.810 E-27) |
| 3 | KM076651 | KC261968 | KC261974 | 5340 | 5831 | RDP (5.322 E-14), GENECONV(1.736 E-12), BootScan (4.687 E-14), MaxChi (60918 E-10), Chimaera (5.871 E-10), SiScan (1.846 E-12) |
| 4 | KM076651 | NC_002052 | HM581940 | 1414 | 3426 | RDP(8.814 E-10), GENECONV(3.503 E-05), BootScan(60615 E-05), MaxChi (1.982 E-12), Chimaera(4.723 E-12), SiScan(2.706 E-19), 3Seq (5.364 E-04) |
| 5 | KM076651 | HM581934 | HM581937 | 7160 | 7501 | RDP (3.967 E-11), GENECONV(1.598 E-09), BootScan (3.775 E-11), MaxChi (4.724 E-06), Chimaera(9.808 E-6), SiScan(1.518 E-06) |
| 6 | KM076651 | KC261953 | KC261974 | 6538 | 6964 | RDP (2.662 E-10), GENECONV(6.428 E-09), BootScan(2.124 E-09), MaxChi(2.068 E-03), Chimaera (1.131 E-03), SiScan(1.007 E-04) |
| 7 | KM076651 | KC261968 | HM581937 | 3987 | 4266 | RDP(7.748 E-04), GENECONV (4.826 E-07), BootScan(4.978 E-09), MaxChi (1.305 E-03), Chimaera (7.713 E-03), SiScan (2.196 E-04) |
| 8 | KM076651 | AB190813 | KC261974 | 1633 | 1839 | RDP (1.483 E-03), MaxChi (1.988 E-03), SiScan (1.222 E-07) |
| 9 | KM076651 | NC_002052 | KC261974 | 4465 | 4732 | GENECONV (1.096 E-05), BootScan (1.845 E-07), SiScan (1.631 E-03) |
| 11 | KM076651 | TSWV WA-USA | KC261968 | 2644 | 3019 | RDP (7.570 E-5), GENECONV(1.692E-03), MaxChi (8.669 E-04), Chimaera(3.732 E-04), 3Seq (3.874 E-04) |
| 12 | KM076651 | HM581940 | HM581934 | 2036 | 2273 | GENECONV (3.866 E- 03), BootScan (6.197 E-04), 3Seq (9.719 E-08) |
